# Grisel’s syndrome in Kawasaki disease

**DOI:** 10.1186/s13023-020-01535-0

**Published:** 2020-09-11

**Authors:** Xiaoliang Liu, Kaiyu Zhou, Yimin Hua, Mei Wu, Lei Liu, Shuran Shao, Chuan Wang

**Affiliations:** 1 Department of Pediatric Cardiology, West China Second University Hospital, Sichuan University, Chengdu, 610041 Sichuan China; 2 Key Laboratory of Birth Defects and Related Diseases of Women and Children (Sichuan University), Ministry of Education Chengdu, Chengdu, 610041 Sichuan China; 3 Key Laboratory of Development and Diseases of Women and Children of Sichuan Province, West China Second University Hospital, Sichuan University, Chengdu, 610041 Sichuan China; 4 The Cardiac development and early intervention unit, West China Institute of Women and Children’s Health, West China Second University Hospital, Sichuan University, Chengdu, 610041 Sichuan China; 5 West China Medical School of Sichuan University, Chengdu, 610041 Sichuan China

**Keywords:** Atlantoaxial subluxation, Cervical lymphadenopathy, Neurological impairment, Coronary artery lesions, Children

## Abstract

**Background:**

Approximately 50–70% of patients with Kawasaki disease (KD) could present with cervical lymphadenopathy associated with deep neck inflammation, which may result in Grisel’s syndrome (GS). Given the possibility of neurological impairment owing to GS, it is important to understand the disease profile in KD. Therefore, we carried out this study to investigate this possible complication of KD, with the aim of improving pediatricians’ recognition and awareness.

**Methods:**

Patients with KD complicated by GS in our hospital were retrospectively recruited for our study. The profiles of patients with GS (*n* = 10) were compared to those patients without GS (*n* = 1254). All the available literature describing these complications of KD was reviewed.

**Results:**

The incidence of GS in KD was 0.6% in our population. Compared to patients without GS, KD patients with GS were older, presented with a significantly lower male:female ratio, and a higher incidence of cervical lymphadenopathy, a higher level of neutrophil count, and erythrocyte sedimentation rate. Ten articles reporting 14 KD patients with GS were reviewed. Of the total 24 patients, GS affected 7 males and 17 females, aged from 3.5 to 9 years old. Encouragingly, no delayed diagnosis and treatment of KD was found, and all patients received conservative therapy for GS, without intravenous immunoglobulin resistance, coronary artery lesions, and neurological impairment.

**Conclusions:**

GS is a rare complication of KD with an incidence of 0.6%, predominantly affecting older, female children. The overall outcome of this disorder in KD was satisfactory with conservative therapy. Pediatricians, especially pediatric surgeons, should recognize and be aware of this possible complication of KD to avoid misdiagnosis and overtreatment.

## Background

Kawasaki disease (KD) is an acute systemic vasculitis of unknown origin that predominately affects children aged 6 months to 5 years old [1]. Apart from the classic clinical features, KD can also affect multiple organs and tissues. Involvement of the pulmonary, gastrointestinal, neurological, and urinary systems has been well recognized and reported in patients with KD [2–7].

However, Grisel’s syndrome (GS), a rare, non-traumatic, atlantoaxial subluxation resulting from any inflammatory condition of the upper neck and otolaryngologic procedures, is rarely reported as a possible complication of KD [8–10]. Indeed, the presence of cervical lymphadenopathy associated with deep neck inflammation, occurring in 50–70% of patients with KD, could lead to parapharyngeal and retropharyngeal edema and nonsuppurative phlegmon [11], and in turn possibly predispose patients to GS. Several cases of KD with GS are described in the literature [8, 9, 12–19]. Given the possibility of life threatening neurological impairments owing to GS, such as radiculopathy or paralysis [20], it is of utmost importance to understand the disease background, course, clinical features, diagnostic issues, and prognosis. This will avoid misdiagnosis and delayed treatment. Nevertheless, little is collectively known about this disorder in KD.

In the present study we report 10 cases of KD with GS, to systemically determine the profile of this possible complication by comparing KD patients with GS to those without GS, and analyze the literature describing this complication.

## Methods and materials

We retrospectively reviewed data for patients diagnosed with KD between January 2015 and December 2019 at our hospital. Informed written consent was obtained from the guardians following a full explanation of the nature of the study. The University Ethics Committee approved the study on Human Subjects at Sichuan University. All research was performed in accordance with relevant guidelines and regulations.

The diagnosis of KD was confirmed by two pediatricians (including at least one KD specialist) in accordance with the 2004 American Heart Association Recommendations for KD [21]. In total, 1582 patients were diagnosed with KD upon admission. Those who had received intravenous immunoglobulin (IVIG) treatment in other medical facilities (*n* = 196), or who did not receive IVIG treatment prior to 10 days from fever onset (*n* = 29), were initially excluded. Another 30 patients were also excluded due to a lack of laboratory data prior to IVIG treatment. Additionally, we excluded 63 patients because the follow-up results were incomplete. Finally, 1264 patients were enrolled for analysis, including ten patients with GS and 1254 patients without GS. To explore the background, clinical course, clinical features, diagnostic and treatment issues, and prognosis of GS in KD, the profiles of the patients with GS were compared to those without GS.

The diagnosis of GS was decided upon by the combination of a clinical assessment, and the presence of rotational atlantoaxial subluxation identified by X-rays and/or computed tomography (CT) scans of the cervical spine and confirmed by a pediatric surgeon. Traumatic and neurologic causes were excluded in all patients with GS [10, 22, 23]. The Fielding and Hawkins system was used to classify the displacement of the atlas with respect to the odontoid in patients with GS [24]. The therapeutic strategy for GS is based on the Fielding-Hawkins classification, including non-steroidal anti-inflammatory drugs, physiotherapy, analgesics and surgery [25].

All the patients received the same treatment program after the diagnosis of KD was established. IVIG (2 g/kg given as a single intravenous infusion) and aspirin (30–50 mg/kg/day) were administered until 48–72 h after fever cessation. After the patient’s fever had resolved, the dose of aspirin was then decreased to 3–5 mg/kg/day, and continued for the next 6–8 weeks. The aspirin was discontinued until the patient showed no evidence of coronary abnormality [26]. If the patient had recurrent or persistent fever for more than 36 h after the IVIG administration, a second IVIG (2 g/kg) was administered. Furthermore, methylprednisolone and/or prednisone were administered as an additional treatment if the patient had recurrent or persistent fever after the second IVIG administration. IVIG-resistance was defined by a persistent or recurrent fever (temperature ≥ 38.0 °C orally) or other clinical signs of KD at least 36 h, but not longer than 7 days, after the initial IVIG.

Coronary artery lesions (CALs) were defined on the normalization of dimensions for body surface area as Z scores (standard deviation units from the mean, normalized for body surface area) as follows: no involvement (z score < 2.0), dilation (z score ≥ 2.0 to < 2.5), aneurysm (z score ≥ 2.5; z ≥ 10 for giant aneurysm) coronary arteries on the basis of the maximal internal diameters of the right coronary artery, left anterior descending artery, and left circumflex coronary artery. In accordance with our institutional protocol, patients underwent standardized echocardiography by two pediatric ultrasonologists during the acute phase, and 6–8 weeks later during follow-up evaluations in the cardiology clinic, until the CALs had resolved.

In addition, all available literature describing this complication in patients with KD were reviewed after a computerized search. This research was performed (without language restriction) via PubMed, Google Scholar, and Scopus, combining the terms “Kawasaki disease AND (Grisel’s Syndrome or atlantoaxial subluxation),” with any filters. Articles written in Chinese were searched for using the same keywords on the China Medical website. Further, all references listed within the articles were subsequently searched for other related articles. Any other relevant literature was also identified by the citation tracker. An eligible article was included if it reported cases with full clinical data consistent with the diagnostic criteria of GS and KD. With regards to the literature search, two articles in English were found, including two patients with KD complicated by GS [8, 9]. One article including three patients was written in Chinese [19]. Another one article was excluded for lacking the baseline information and the detail of clinical features required. After searching the listed references for other related articles, another six relevant studies [12, 14–18] in Japanese were identified by the citation tracker, including eight patients. These Japanese articles were further translated by Chinese-Japanese bilingual biomedical scientists. Six articles were single case reports and four were case series. The following epidemiologic and clinical variables were evaluated for each case: sex, age, duration before diagnosis, duration of treatment, the inflammatory condition of the upper neck, side of lymphadenopathy, side of rotatory dislocation, fielding classification, CALs, treatment, and outcome (Table 1).

**Table 1:** The summary of Grisel’s syndrome in Kawasaki disease

Patient	Author, country	Age(years), Sex	Duration before diagnosis	Clinical presentation	Lymphadenopathy	Fielding classification	Rotatory dislocation	Treatment	Duration treatment	CALs
1	Igarashi,1989 [12] Japan	7, F	7 days	Tilted position	Left	–	Left	Conservative	2 months	None
2	Kaketa, 2004 [14] Japan	5, F	5 months	Torticollis	–	–	Left	Conservative	3 months	–
3	Konishi, 2007 [15] Japan	4, F	11 days	Torticollis	Left	Type I	Left	Conservative	13 days	–
4	Konishi, 2007 [15] Japan	4, F	4 days	Torticollis	Left	Type I	Left	Conservative	8 days	–
5	Oda, 2009 [17], Japan	9, M	13 days	Torticollis	Right	Type I	Right	Conservative	3 days	None
6	Oda, 2009 [17], Japan	6, F	15 days	Torticollis	Right	Type I	Right	Conservative	15 days	None
7	Oda, 2009 [17], Japan	6, M	14 days	Torticollis	Bilateral	Type I	Left	Conservative	7 days	None
8	Tashiro, 2011 [18], Japan	6, F	3 days	–	–	–	Right	Conservative	–	–
9	Nozaki, 2013 [9], Japan	5, F	22 days	Tilted position	Left	Type I	Left	Conservative	2 months	None
10	Wood [8], 2013, Australia	8, F	8 days	Torticollis Neck pain and stiffness	Left	–	Left	Conservative	3 months	None
11	Tian LP [13], 1995, China	6, F	9 days	Torticollis Neck pain and stiffness,	Bilateral	Type I	Left	Conservative	25 days	None
12	Wang Ce, 2018 [19], China	5, M	–	Neck pain and stiffness,	–	–	–	Conservative	3 days	None
13	Wang Ce, 2018 [19], China	5, M	–	Neck pain and stiffness	–	–	–	Conservative	6 days	None
14	Wang Ce, 2018 [19], China	5, M	–	Neck pain and stiffness	–	–	–	Conservative	7 days	None

### Statistical analysis

All data were analyzed using SPSS version 21.0 (SPSS Inc. Chicago, IL, USA). Quantitative data are presented as the median with the 25th and 75th percentiles (interquartile range [IQR]) in square brackets, while qualitative data are expressed as n/%. Shapiro-Wilk and homogeneity of variance tests were used to confirm that the quantitative data from the different groups exhibited a normal distribution with regards homogeneity of variance. Differences in quantitative data between patients with and without GS were assessed using independent samples t-tests or Mann–Whitney U-tests. To assess nonparametric dichotomous and non-dichotomous variables, we used contingency tables/Fisher’s exact tests and regression analyses with dummy variables, respectively. The level of statistical significance was set at *P* < 0.05 (two-tailed).

## Results

**Fig. 1:**
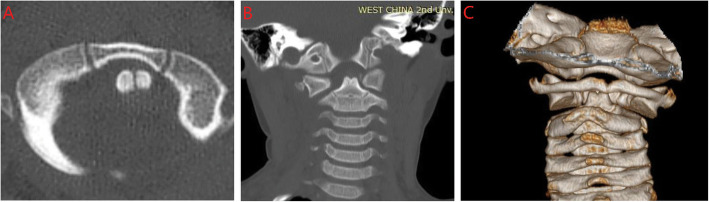
An axial computed tomography (CT) scan shows atlantoaxial rotatory subluxation (**a** and **b**). A three-dimensional CT scan showing subluxation of the C1-C2 cervical tract (**c**)

Of the 1582 KD patients recruited in our study, ten cases of GS were identified as a complication of KD, with the incidence of 0.6% (10/1582). In patients with KD presenting cervical lymphadenopathy, its constituent ratio referred to 1.5% (10/657). It affected two males and eight females, aged from 3.5–7.3 years old. Two patients (P2, P5) were admitted via the Pediatric Surgery Department for the initial complaint of neck pain and tilted position, before fever onset. P1 was admitted via the Neurological Department with a primary suspected diagnosis of lymphnoditis and aseptic meningitis. Another seven patients were admitted via the Cardiology Department for suspected KD with a primary diagnosis of lymphnoditis (*n* = 5), tonsillitis (*n* = 1), and lymphnoditis and GS (*n* = 1). The time before the diagnosis of GS and KD ranged from 2 to 10 days and 5–9 days, respectively. The typical clinical features of KD presented with persistent fever (*n* = 10), bilateral non-exudative conjunctivitis (*n* = 8), erythema of the lips and oral mucosa (*n* = 8), changes in the extremities (*n* = 3), rash (*n* = 5), and cervical lymphadenopathy (*n* = 10). The mean size of lymphadenopathy was 2.9 cm (range: 2.0–5.0 cm). Six patients out of ten (60.0%) with GS were diagnosed with incomplete KD. The symptoms of GS were neck pain (*n* = 9) and torticollis (*n* = 10). Among them, the CT scans of the cervical spine identified four of Fielding-Hawkins classification type I, two of type II, and four of type III, respectively (Fig. 1). The patients underwent the conservative treatment of a cervical collar for 7 days to 3 months, and resolved without neurological impairment. No patients received surgical therapy. All patients received timely and prompt treatment with IVIG and aspirin within 10 days from fever onset (Table 2).

**Table 2:** The clinical characteristics of Grisel’s syndrome in patients with Kawasaki disease

Patient	1	2	3	4	5	6	7	8	9	10
Age(years), Sex	6.6, F	5.7, M	3.7, F	7.2, F	6.3, F	6.2, M	7.3, F	3.5, F	4.5, F	5.2, F
Initial admitted Department	Neurology	Surgeon	Cardiology	Cardiology	Surgeon	Cardiology	Cardiology	Cardiology	Cardiology	Cardiology
Primary diagnosis	Lymphnoditis, Aseptic meningitis	Lymphnoditis	Lymphnoditis	Lymphnoditis	Lymphnoditis, GS	Lymphnoditis, GS	Lymphnoditis	Lymphnoditis	Tonsillitis	Lymphnoditis
Days before the diagnosis of GS	5	5	2	6	4	8	10	4	7	8
Days before the diagnosis of KD	7	8	5	6	5	8	6	6	7	6
Fever days before IVIG treatment	7	9	5	6	6	9	7	6	9	7
Principal clinical features of KD	1, 2, 3, 6	1, 2, 5, 6	1, 3, 5, 6	1,3, 6	2, 3, 6	1,2,3,4, 6	1,2,3,5,6	1,2,3,4,5,6	1, 2, 4, 5, 6	1, 2, 3, 6
Incomplete KD	+	+	+	+	+	–	–	–	–	+
IVIG resistance	–	–	–	–	–	–	–	–	–	–
CALs	–	–	–	–	–	–	–	–	–	–
**Clinical features of GS**										
Torticollis/Neck pain	+/+	+/+	+/+	+/+	+/+	+/+	+/+	+/+	+/+	+/−
Lymphadenopathy, size(mm)	R^a^, 4.5*5.0	L^a^, 2.5*2.5	L, 3.5*3.0	L, 2.0*2.0	L, 1.5*2.0	L, 1.5*2.5	R, 1.9*3.6	L, 1.8*2.7	R, 2.5*1.6	L, 3.0*4.0
Fielding classification	Type I	Type III	Type I	Type I	Type III	Type II	Type II	Type I	Type III	Type III
Rotatory dislocation	Right	Left	Left	Left	Right	Left	Left	Left	Left	Left
Treatment	Conservative	Conservative	Conservative	Conservative	Conservative	Conservative	Conservative	Conservative	Conservative	Conservative
Duration treatment	1 month	2 months	14 days	7 days	1.5 months	21 days	21 days	7 days	3 months	2.5 months

Principal clinical features of KD:1. Fever for at least 5 days; 2. Bilateral bulbar conjunctival injection without exudate; 3. Changes in lips and oral cavity;4. Changes in extremities; 5. Polymorphous exanthem; 6. Cervical lymphadenopathy

^a^R, right side; L, left side

A detailed comparison of the clinical data between KD patients with and without GS was presented in Table 3. KD patients with GS were older (median age: 5.9 vs 2.2 years, *p* < 0.001), presenting with a significantly lower male ratio (20.0% vs 58.3%, *p* = 0.021) and a higher incidence of cervical lymphadenopathy (100% vs 51.6%, *p* = 0.002). In terms of the clinical features, the rate of IVIG resistance and CALs, there were no significantly differences between the two groups (all *p* > 0.05). The development of CALs and IVIG resistance did not occur in KD patients with GS. Prior to the initial IVIG treatment, the neutrophil count and erythrocyte sedimentation rate were significantly higher in patients with GS than patients without GS (all *p* < 0.05).

**Table 3:** Comparison of clinical data between the groups of Kawasaki disease (KD) with and without Grisel syndrome (GS)

	KD with GS (***n*** = 10)	KD without GS (***n*** = 1254)	***p*** value
Male, n (%)	2(20.0)	731(58.3)	0.021
Age, years	5.9(4.3–6.7)	2.2(1.2–3.6)	<0.001
**Clinical manifestations**			
Rash	5(50.0)	939(74.9)	0.134
Edema & erythema of the extremities	3(30.0)	715(57.0)	0.111
Bilateral bulbar conjunctive injection	8(80.0)	1138(90.7)	0.226
Erythema of oral and pharyngeal mucosa	8(80.0)	1142 (91.1)	0.256
Cervical lymphadenopathy	10(100.0)	647(51.6)	0.002
Fever duration before IVIG administration, days	6.0(4.8–7.3)	6.0(5.0–7.0)	0.916
Incomplete KD	6(60.0)	460(36.7)	0.186
IVIG resistance	0(0.00)	188(15.0)	0.374
Coronary artery lesions	0(0.00)	125(10.6)	0.292
**Laboratory examinations**			
White blood cell, ×10^9^/L	14.6(12.5–18.8)	14.0(11.0–17.4)	0.744
Neutrophil count, %	79.9(77.2–91.7)	68.3(58.0–78.6)	0.001
Hemoglobin, g/L	108.0(99.3–117.5)	109.0(102.0–116.0)	0.737
Platelet count, ×10^9^/L	313(268–350)	325(267–395)	0.344
C-reactive protein, mg/L	111.0(69.5–155.5)	76.0(45.0–114.0)	0.155
Erythrocyte sedimentation rate, mm/h	102.0(59.0–124.0)	66.0(46.0–87.0)	0.018
Aspartate aminotransferase, U/L	34.5(27.0–56.5)	34.0(26.0–52.0)	0.573
Alanine aminotransferase, U/L	25.5(13.0–39.0)	37.0(21.0–79.0)	0.332
Albumin, g/L	36.0(31.7–42.3)	37.5(34.1–40.9)	0.667
Total bilirubin, umol/L	9.2(7.1–15.5)	6.3(4.0–10.0)	0.858
Creatinine, umol/L	30.0(25.0–41.5)	28.0(23.0–33.0)	0.839
Urea nitrogen, umol/L	2.7(2.3–4.9)	2.9(2.2–4.0)	0.429
Serum sodium, mmol/L	138.2(135.1–138.2)	136.9(134.0–139.0)	0.880
Potassium, mmol/L	4.0(3.7–4.1)	4.1(3.7–4.5)	0.396

The data are presented as the median with the 25th and 75th percentiles in square brackets for continuous variables and as n/% for qualitative data as appropriate

## Discussion

In children, GS could involve patients with KD due to the neck involvement presenting with cervical lymphadenopathy, but is rarely reported. To date, only 14 cases have been described worldwide in patients with KD from 1989 to 2019 [8, 9, 12–19]. To our knowledge, this study has the largest sample size and is the first to systematically report the profiles of GS in patients with KD. As the third report in the English literature [8, 9], we summarize the epidemiology, clinical features, and outcomes of this disorder as a complication of KD.

Ten cases of GS were found in our population, with a low incidence of 0.6%, accounting for 1.5% of patients with KD exhibiting cervical lymphadenopathy. This disorder in KD is more likely to affect older children, is dominant in females, and has an uneven racial distribution most often affecting Asians, especially the Chinese and Japanese. In comparison to patients without GS, only two parameters (neutrophil count and erythrocyte sedimentation rate) were significantly different in KD with GS. This disorder did also not increase the risk of CALs in KD, suggesting a less severe inflammatory burden. Encouragingly, the overall outcome of this disorder in KD was satisfactory. Almost all the children had a timely diagnosis and adequate treatment, without IVIG resistance or neurological impairment. The conservative therapy appeared to be enough for this disorder in KD.

After the literature review, a total of 24 cases of GS were identified in patients with KD including ten patients from our population. The mean age of these patients was 6 years old (range 3.5 to 9 years old), affecting seven males and 17 females (M/F ratio was 0.41). The mean duration before the identification of this disorder in KD was 9 days (range 3 to 22 days). However, the longest duration (P2) was 5 months. Another three patients reported by Wang [19] did not describe the duration before the diagnosis of GS. Besides patient 8 (P8) whose symptoms were not mentioned, all others complained of tilted position, stiffness, or torticollis, while neck pain was described in 14 (60.9%) patients. All patients presented with cervical lymphadenopathy: 12 on the left side, five on the right, and two bilateral. Five were not described. Of the patients, 11 presented with Fielding-Hawkins classification type I, two with type II, and four with type III. The previous reported cases did not clarify whether patients with KD received IVIG within 10 days. The occurrence of IVIG resistance and CALs were not noted in the studies. The patient’s rotatory dislocation recovered to normal after receiving conservative treatment for GS with a duration of 3 days to 3 months, without neurological impairment.

The presence of lymphadenopathy was the initial and predominant symptom in these patients. Primarily misdiagnosed as lymphnoditis, it gradually evolved into GS. The mean size of the cervical lymphadenopathy was 2.9 cm, which was greater than in the general KD population (1.5 cm) [27]. In accordance with patients with upper respiratory tract infection (URTI) regarded as a common cause of GS [28, 29], we found this disorder in KD prone to involve older children (median age: 5.9 years) than in the general KD population. This is associated with a high prevalence of cervical lymphadenopathy in older children with KD [30], which is partly in line with the prior findings by Nozaki [9]. More importantly, with regards to the alar and transverse ligaments, these are characteristically lax in children and the increasing activity of these ligaments as the child ages could also worsen this condition. In our patients with GS, the size of the cervical lymphadenopathy in KD was remarkably larger than that of the general KD population, which might significantly contribute to the development of GS. In addition, two of our patients were referred to the Pediatric Surgery Department for cervical lymphadenopathy as the initial symptom. Regrettably, the pediatric surgeon failed to recognize KD with GS, although the familiar features of KD gradually presented. These findings suggest that pediatricians, especially pediatric surgeons, should identify the possibility of GS as a complication of KD, since conservative therapy appears to be enough for this group of children and the general outcomes are satisfactory with a timely diagnosis and treatment.

However, some differences are apparent in patients with GS when comparing patients with KD to those with URTI. Firstly, there is an uneven population distribution of GS in patients with KD. All the available literature, as well our study, show Asians are more likely be affected, with only one patient of western origin. This might be associated with the prevalence of cervical lymphadenopathy in different ethnic groups with KD. In patients with KD from Japan and China, the incidence of cervical lymphadenopathy is 50–70% [31] and 54.6% [32], respectively, which was relatively higher than that of western countries (43%) [33]. Secondly, the M/F ratio in the general KD population is 1.31–1.62, and it decreases as the patient age increases. However, no female dominance has been observed [34]. In accordance with the prior findings by Fath et al., we found that GS was more likely to involve females, but that female dominance was not found in patients with GS resulting from other inflammatory conditions of the upper neck, namely tonsillitis and pharyngotonsillitis [28, 35]. Regrettably, the cause of female dominance in patients with KD complicated by GS remains unknown. Finally, the mean time from disease onset to the diagnosis of GS (30 days) in other inflammatory diseases was much longer than that of patients with KD (just 9 days). The shorter duration in KD might account for the satisfactory prognosis, without requiring further therapy such as surgery, or any neurological impairment.

This study had a potential limitation with regards the small sample size. This is inevitable considering the data were collated from only one center, and GS in KD is a distinctly rare presentation of this disorder. Moreover, the phenomenon may be transient in many more patients with KD. This leaves it easily ignored and not recognized by pediatricians due to the short duration, as well as spinal imaging not being within the routine examination during the acute phase of KD. Additionally, literature written in other languages might have been missed, although our computerized search had no filter or language restrictions.

## Conclusions

GS is a rare complication of patients with KD with an incidence of 0.6%. It seems to mainly affect older, female, Asian children. The presence of lymphadenopathy was the initial and predominant symptom in these patients. Encouragingly, the overall outcome was satisfactory. Almost all the children had a timely diagnosis and adequate treatment, even without IVIG resistance, neurological impairment, or CALs. The conservative therapy appeared to be enough for this disorder in KD. This information was important when counseling the patient’s parents and may relieve any anxiety they might have. Pediatricians, especially pediatric surgeons, should recognize and be aware of this possible complication of KD to avoid misdiagnosis and overtreatment.

## Data Availability

Not applicable.

## Abbreviations

GS: Grisel’s Syndrome
KD: Kawasaki Disease
IVIG: Intravenous immunoglobulin
CT: Computed tomography
CALs: Coronary artery lesions
URTI: Upper respiratory tract infection
